# Occurrence and Correlates of Suicidal Thoughts Among Young Autistic Users of a Mental Health App

**DOI:** 10.1002/aur.70251

**Published:** 2026-04-15

**Authors:** T. L. Procyshyn, E. M. Weir, M. Pelton, T. Chikaura, H. Hodges, K. Comley, S. Godson, C. Allison, S. Baron‐Cohen

**Affiliations:** ^1^ Department of Psychiatry, Autism Research Centre University of Cambridge Cambridge UK; ^2^ Tellmi Health Foundry London UK

**Keywords:** autism, depression, mental health, self‐harm, suicide

## Abstract

Young autistic people experience disproportionately high rates of mental health challenges, yet little is known about factors associated with suicidality in this group. This study leveraged anonymous self‐report data to identify correlates of suicidal thoughts among 365 young users (aged approximately 11–25 years) of a mental health app in the United Kingdom with an autism diagnosis or self‐identifying as autistic. The presence of suicidal thoughts was assessed as a binary item. Binary logistic regression was used to explore correlates of suicidal thoughts across three domains: mental health‐related symptoms, autism‐related factors, and adverse life events/experiences. The final model included all significant correlates from domain‐specific models alongside demographic factors. Suicidal thoughts were reported by 63% of participants, with similar rates across age groups. The final model accounted for nearly 50% of variability in the presence of suicidal thoughts (*R* ^2^_Nagelkerke_ = 0.48, *p* < 0.001). Self‐harm and depression showed the strongest positive associations (odds ratio, OR and [95% confidence interval] = 6.41 [3.62, 11.35] and 4.58 [2.51, 8.35], respectively), followed by a history of physical abuse (OR = 3.01 [1.20, 7.56]) and a transgender/gender‐diverse identity (OR = 2.13 [1.11, 4.10]). Personal use of the term “neurodiversity” was associated with a lower likelihood of suicidal thoughts (OR = 0.45 [0.24, 0.83]). These findings contribute to evidence of high rates of suicidal thoughts among young autistic people and associations with self‐harm, depression, a history of abuse, and a gender minority identity. Self‐identification with the term neurodiversity emerged as a potential protective factor, although this novel finding warrants further research. Overall, this study highlights the importance of access to autism‐adapted mental health care, addressing trauma and identity‐related stressors, and fostering belonging and connection as part of comprehensive suicide prevention strategies for autistic youth.

## Summary

This study analyzed data from 365 young autistic people in the United Kingdom who were users of a mental health app to examine how common suicidal thoughts were and what factors were associated with them. Suicidal thoughts were reported by 63% of participants and were most strongly linked to depression, self‐harm, history of physical abuse, and having a gender minority identity. As this was not a population‐based study, our sample is likely to experience higher levels of mental health difficulties than young autistic people in general. These findings stress the need for accessible autism‐adapted mental health care, addressing trauma and identity‐related stressors, and fostering belonging and connection as part of comprehensive suicide prevention strategies for autistic youth.

## 1 Background

Autistic people experience higher rates of suicidal ideation (thinking about suicide), suicide attempts (intentionally engaging in behavior with intent to end one's life), and death by suicide compared to the general population (Brown et al. 2024; Newell et al. 2023). Recent findings are particularly concerning for young autistic people. The most recent meta‐analysis of suicidality among autistic people aged < 25 years estimated that 25% experience suicidal ideation, 8% attempt suicide, and 0.2% die by suicide (O'Halloran et al. 2022). These rates are substantially higher than estimates for general adolescent populations of 12% for suicidal ideation and 4% for suicide attempts (Nock et al. 2013). A study of USA hospital emergency department visit data over an 8‐year period further underscores this issue, with suicidal ideation or intentional self‐inflicted injury present in 5.1% of young autistic people compared to 1.2% of the non‐autistic comparison group (Cervantes et al. 2023).

Despite growing awareness of mental health challenges among autistic people, the complex interaction of factors underlying this risk is not yet fully understood. Prior studies of suicidality among autistic youth have identified many of the same correlates reported for autistic adults and the general population, including depression, anxiety, and history of trauma (Horowitz et al. 2018). An exploratory study of autistic youth aged 6–19 found that autism‐related characteristics (i.e., social‐communication difficulties, restricted/repetitive behaviors), co‐occurring conditions (affective and conduct disorders), and sociodemographic factors (IQ, parental education) were associated with suicidality (McDonnell et al. 2020). The cumulative pressures of adolescence, such as developmental transitions and the need to navigate increasingly complex social environments, may heighten vulnerability to mental health distress among young autistic people. Consistent with this, perceived loneliness, externalizing behaviors, bullying, and gender‐identity related stigma have been associated with increased suicidality in autistic youth, whereas resilience has been found to be protective (La Buissonnière Ariza et al. 2022; O'Halloran et al. 2022).

Nevertheless, notable research gaps remain. Factors associated with mental health challenges among autistic adults—such as camouflaging of autistic traits (Cassidy et al. 2020) or being gender nonconforming (Mournet et al. 2024)—have received limited attention in younger samples. Similarly, factors associated with suicidality in non‐autistic adolescent samples, such as self‐harm, substance use, and sleep problems (Mars et al. 2019), have not been specifically examined in autistic youth.

To better characterize the occurrence and correlates of suicidal ideation among young autistic people (diagnosed or self‐identifying), the present study draws on anonymous self‐report data from users of a mental health app for young people in the United Kingdom. While such a sample is subject to sampling bias and likely enriched for people with mental health difficulties, this design addresses a limitation of many prior studies that rely on informant report (e.g., parents, medical records), which likely underreport suicidality and overlook salient psychosocial stressors experienced by young people (Newell et al. 2023; O'Halloran et al. 2022).

Accordingly, this study aims to (1) examine the proportion of young autistic people in our cohort experiencing suicidal thoughts and (2) examine associations between suicidal thoughts and demographic, clinical, and social factors. By focusing on users of a mental health support app, this study seeks to inform clinical care tailored to young autistic people engaging with digital mental health services.

## 2 Methods

### 2.1 Study Design

This study involves secondary analysis of anonymous data collected as part of a user survey for the Tellmi app (www.tellmi.help/). Tellmi is a UK‐based, professionally moderated peer‐support mobile app designed to support the well‐being of young people (typically aged 11–25 years). Users can anonymously post descriptions of personal experiences or difficulties and receive supportive responses from peers. All posts are categorized by topic (e.g., anxiety, autism, family conflict). Clicking on topic tags filters the feed so that users are talking to a community of similarly aged individuals (within 2 years) who are dealing with the same challenges. Unlike social media platforms, Tellmi provides a structured framework in which all user‐generated content is reviewed by trained adult moderators to prevent bullying and exposure to harmful materials. Any high‐risk content is flagged by moderators who transfer the post to a qualified counselor who responds privately and, when necessary, will initiate further protective action. An integrated signposting directory that can be filtered by topic provides direct links to crisis support (e.g., NHS, Childline, SHOUT), specialist support, and postcode‐matched local services, as well as tools, apps, and psychoeducational resources. Tellmi meets the NICE evidence standard for a tier B digital health technology.

As Tellmi has recognized that a large proportion of users are autistic,^1^ they launched a targeted survey for users with an autism diagnosis or suspected autism between February and June 2023. The survey was co‐designed with autistic Tellmi users, who reviewed, adjusted, and approved the final survey items. Although the primary aim of the survey was to better understand use of Tellmi to inform service improvements, survey items also included demographics (age, gender, ethnicity), co‐occurring conditions, mental health challenges (including suicidal thoughts), school‐ and family‐related challenges, and challenges relating to autism. Participants also had the option to enter free‐form responses to open‐ended questions about their mental health and what they like about the app. The survey was advertised through the Tellmi app and open to all users with an autism diagnosis or those who suspect they are autistic. Participants provided informed consent and were informed that the survey responses would be used for research purposes only. This secondary analysis of anonymous data from Tellmi was favorably reviewed by the Cambridge Psychology Research Ethics Committee (PRE.2024.034).

### 2.2 Inclusion Criteria

A total of 829 participants initiated the survey. We excluded participants who did not provide consent (*n* = 4), completed an early version of the survey without demographic items (*n* = 16), or did not progress beyond the initial demographic items (*n* = 198). We also excluded participants who responded to the initial survey question “Do you believe you are autistic?” as no (*n* = 25) or unsure (*n* = 221).^2^ Subsequent questions clarified if participants had a formal autism diagnosis or were seeking one.

The final sample comprised 365 participants who believe they are autistic, including 122 with a formal autism diagnosis, 206 without an autism diagnosis (72 of whom reported being on an autism assessment waiting list), and 37 who were unsure whether they had an autism diagnosis.

### 2.3 Measures

As noted above, the Tellmi user survey was not designed to explore suicidality as an outcome. The following items were selected as relevant to the present analysis:

#### 2.3.1 Suicidality and Mental Health‐Related Symptoms

Participants were presented a list of health‐related conditions and symptoms and asked “Do you experience any of the following? Select as many as are relevant to you.” This list included suicidal thoughts, anxiety, panic attacks, depression, self‐harm, and sleep problems. These were treated as a binary variable where participants selected the item (Yes) or did not (No).

#### 2.3.2 Demographics

Participants reported their age range (11–13, 14–16, 17–18, or 19+), details of their autism diagnosis, and ethnicity. The survey included two items related to gender identity: (i) “What is your gender identity?” and (ii) “Is your gender the same as the one you were assigned at birth?.” Participants who responded female/yes were categorized as cisgender girl/woman, participants who responded male/yes were categorized as cisgender boy/man, and participants who responded with any other combination (e.g., nonbinary, agender, gender questioning, transgender, multiple gender identities) were categorized as gender minority.

#### 2.3.3 Autism‐Related Factors

Participants reported the extent to which they experienced autism‐related differences relating to the domains of social interactions, communication, sensory processing, social camouflaging/masking, and preference for sameness; responses Yes, Sometimes, or No were coded as 3, 2, or 1, respectively. Participants were also asked “Do you know what the term ‘neurodiversity’ means?” and “Do you use the term yourself?” (recorded as Yes/No).

#### 2.3.4 Adverse Life Events/Experiences

Participants were presented lists of adverse life events and asked “Have you experienced any of the following? You can select more than one.” The lists included sexual abuse, physical abuse, drug and alcohol abuse, family difficulties, bullying, and loneliness. These were treated as a binary variable where participants selected the item (yes) or did not (no).

### 2.4 Analysis

Descriptive data are reported as count (*n*) or proportion (%). Comparisons of binary outcomes between groups were assessed using the *χ* ^2^ test. Correlates of suicidal thoughts were explored through a series of binary logistic regression models controlling for demographic factors (age range, formal autism diagnosis, gender minority identity, and ethnic minority identity). Model 1 included mental health‐related symptoms, Model 2 included autism‐related factors, and Model 3 included negative life experiences. The final model included demographic factors plus all variables from Models 1–3 that remained statistically significant after applying false discovery rate (FDR) correction. The absence of significant collinearity between independent variables was confirmed using the variance inflation factor.

## 3 Results

### 3.1 Prevalence of Suicidal Thoughts

The majority of participants (63%) reported suicidal thoughts. Table 1 presents participants' demographic and the proportions reporting suicidal thoughts by demographic subgroup. The proportion of participants reporting suicidal thoughts did not differ significantly between age groups (*χ* ^2^ = 0.76, *p* = 0.86), individuals with or without/unsure of a formal autism diagnosis (*χ* ^2^ = 0.88, *p* = 0.35), or by ethnicity (*χ* ^2^ = 0.01, *p* = 0.91). There was a significant relationship between gender identity and suicidal thoughts (*χ* ^2^ = 15.7, *p* < 0.001), with a greater proportion of gender minority individuals reporting suicidal thoughts compared to cisgender girls/women or cisgender boys/men.

**TABLE 1:** Demographic characteristics of autistic participants (*n* = 365) and proportion experiencing suicidal thoughts across subgroups.

	*n* (%)	Suicidal thoughts	
		Absent, *n* (%)	Present, *n* (%)
Age range			
11–13	43 (11.8%)	17 (39.5%)	26 (60.5%)
14–16	168 (46.0%)	60 (35.7%)	108 (64.3%)
17–18	77 (21.1%)	31 (40.3%)	46 (59.7%)
19+	75 (20.5%)	26 (34.7%)	49 (65.3%)
Prefer not to say/NA	2 (0.5%)	2 (100.0%)	0 (0%)
Autism diagnosis			
Formal diagnosis	122 (33.4%)	41 (33.6%)	81 (66.4%)
No formal diagnosis	206 (56.4%)	83 (40.3%)	123 (59.7%)
Not sure	37 (10.1%)	12 (32.4%)	25 (67.6%)
Gender identity			
Cisgender girl/woman	197 (54.0%)	83 (42.1%)	114 (57.9%)
Cisgender boy/man	36 (9.9%)	20 (55.6%)	16 (44.4%)
Gender minority^a^	129 (35.3%)	32 (24.8%)	97 (75.2%)
Prefer not to say/NA	3 (0.8%)	1 (33.3%)	2 (66.7%)
Ethnicity			
White	317 (86.8%)	118 (37.2%)	199 (62.8%)
Minority ethnicity^b^	42 (11.5%)	16 (38.1%)	26 (61.9%)
Prefer not to say/NA	6 (1.6%)	2 (33.3%)	4 (66.7%)

^a^ Includes individuals reporting their gender identity as nonbinary, transgender, questioning, agender, or self‐describing.

^b^ Includes individuals reporting their ethnicity as Asian, African, multiple ethnicities, or other.

### 3.2 Correlates of Suicidal Thoughts

Correlates of suicidal thoughts were explored through a series of binary logistic regressions with the presence (vs. absence) of suicidal thoughts as the dependent variable, controlling for age range, formal autism diagnosis, gender minority identity, and ethnic minority identity (Table 2). Due to missing data for at least one demographic item for 9 participants, the sample for these analyses comprised 356 individuals. Model 1, which included self‐reported mental health symptoms, showed significantly higher odds ratios of suicidal thoughts among participants reporting depression and self‐harm. Model 2, which included autism‐related factors, showed that greater sensory differences and social masking of autistic traits were associated with a significantly higher odds ratio of suicidal thoughts, whereas use of the term “neurodiversity” was associated with a significantly lower odds ratio. Model 3, which included adverse life events, showed a significantly higher odds ratio of suicidal thoughts among participants reporting a history of physical abuse and loneliness.

**TABLE 2:** Binary logistic regression models exploring correlates of suicidal thoughts in diagnosed and self‐identifying autistic participants.

	OR (95% CI)	*p*
Model 1: Mental health‐related symptoms^a^		
Anxiety	0.71 (0.20, 1.81)	0.48
Panic attacks	1.55 (0.87, 2.77)	0.14
Depression	4.22 (2.30, 7.74)	< 0.001*
Self‐harm	6.83 (3.92, 11.77)	< 0.001*
Sleep problems	1.65 (0.94, 2.91)	0.082
Model 2: Autism‐related factors^b^		
Social interaction differences	1.85 (1.01, 3.37)	0.045
Communication differences	0.84 (0.58, 1.23)	0.38
Sensory differences	1.97 (1.24, 3.20)	0.006*
Camouflaging/masking	1.74 (1.12, 2.64)	0.010*
Preference for sameness	0.77 (0.49, 1.22)	0.27
Use of term “neurodiversity” vs. not	0.47 (0.28, 0.79)	0.005*
Model 3: Adverse life events/experiences^c^		
Physical abuse	3.74 (1.53, 9.11)	0.004*
Sexual abuse	1.25 (0.67, 2.33)	0.46
Drug/alcohol abuse	1.73 (0.76, 3.97)	0.19
Family difficulties	1.70 (1.00, 2.87)	0.046
Bullying	1.10 (0.64, 1.90)	0.73
Loneliness	3.11 (1.23, 7.87)	0.017

*Note*: All models were adjusted for the following demographic variables: age range, formal autism diagnosis, gender minority identity, and ethnic minority identity.

Estimates represent the log odds of the presence versus absence of suicidal thoughts.

Abbreviations: 95% CI, 95% confidence interval; OR, odds ratio.

^a^ Model 1 overall fit: *R* ^2^_Nagelkerke_ = 0.44, *p* < 0.001.

^b^ Model 2 overall fit: *R* ^2^_Nagelkerke_ = 0.16, *p* < 0.001.

^c^ Model 3 overall fit: *R* ^2^_Nagelkerke_ = 0.18, *p* < 0.001.

* *p* < 0.05 after applying FDR correction.

The final model (Table 3), which included demographic variables and all significant variables from Models 1–3, accounted for nearly 50% of variability in the presence of suicidal thoughts (overall model fit: *R* ^2^_Nagelkerke_ = 0.48, *p* < 0.001). Of the included variables, experiencing self‐harm, depression, a history of physical abuse, and a gender minority identity were associated with significantly higher odds ratios of suicidal thoughts. Again, participants who reported that they personally used the term neurodiversity had a significantly lower odds ratio of suicidal thoughts.

**TABLE 3:** Final model of correlates of suicidal thoughts in diagnosed and self‐identifying autistic participants.

Variables	*β*	OR (95% CI)	*p*
Age range	0.05	1.06 (0.77, 1.45)	0.73
Formal diagnosis vs. undiagnosed/unsure	0.44	1.55 (0.83, 2.89)	0.17
Gender minority vs. cisgender	0.76	2.13 (1.11, 4.10)	0.02*
Ethnic minority vs. White	−0.10	0.91 (0.38, 2.15)	0.82
Depression	1.52	4.58 (2.51, 8.35)	< 0.001*
Self‐harm	1.86	6.41 (3.62, 11.35)	< 0.001*
Sensory differences	0.52	1.69 (0.94, 3.02)	0.08
Camouflaging/masking	0.28	1.32 (0.80, 2.18)	0.28
Use of term “neurodiversity” vs. not	−0.80	0.45 (0.24, 0.83)	0.01*
Physical abuse	1.10	3.01 (1.20, 7.56)	0.02*

*Note*: Estimates represent the log odds of the presence vs. absence of suicidal thoughts.

Model overall fit: *R* ^2^_Nagelkerke_ = 0.48, *p* < 0.001.

Abbreviations: 95% CI, 95% confidence interval; OR, odds ratio.

* *p* < 0.05.

As a sensitivity analysis, the final model was also run with the addition of participants who indicated that they were “unsure” if they believe they are autistic. The results were highly consistent, with a gender minority identity, depression, self‐harm, a history of physical abuse, and personal use of the term neurodiversity significantly correlated with the likelihood of suicidal thoughts (Table S1).

## 4 Discussion

In this study of a large, real‐world sample of young autistic people using a mental health support app, suicidal thoughts were strikingly common. While the proportion of participants in our sample reporting suicidal thoughts (63%) is higher than the pooled prevalence estimate determined by the latest meta‐analysis of suicidality in autistic youth (25%: O'Halloran et al. 2022), it is comparable to that reported by some individual studies. For example, a study of transgender autistic youth aged 14–25 found that 88% had experienced suicidal thoughts during their life (Strauss et al. 2021). According to a survey of parents of autistic youth admitted to a psychiatric hospital, 63% reported that their child experienced periods of several days during which they talked about suicide or death (Horowitz et al. 2018). Thus, the high prevalence in our sample likely arises from a combination of the use of self‐report, enrichment for mental health challenges (i.e., participants are existing users of a mental health app), and demographic factors—particularly the high proportion (35%) of participants reporting a gender minority identity. While the prevalence of suicidal thoughts in our sample was similar across age groups (60.5% age 11–13 years, 64.3% age 14–16 years, 59.7% age 17–18 years), a multi‐national study of autistic children and adults suggests that suicidality increases with age; in the study sample, which was enriched for autistic people experiencing mental health challenges, 88% experienced suicidal ideation and 31% reported a suicide attempt at some point in their life (Schwartzman et al. 2025). The study also found that the average age of first suicide attempt in their sample was 16.6 years, while the youngest age of suicide attempt was just 7 years, underscoring the urgent need to understand suicidality among autistic young people and provide support that can prevent progression to suicidal behaviors among those already experiencing suicidal thoughts.

How to best support autistic youth struggling with suicidality is a challenging question. While it is questioned whether suicide can be predicted and prevented (Paris 2021), understanding contributing factors and barriers to help‐seeking can improve understanding of pathways that lead individuals to a crisis point. A recent study reported that many autistic adults in the United Kingdom do not seek support for suicidality because they do not believe that traditional healthcare services can help them or find these services inaccessible (Procyshyn et al. 2025), underscoring the need for alternative sources of mental health support, such as digital health technologies. Insights into risk factors and service barriers can jointly inform the development of mental health services and shape public health policies aimed at reducing suicide rates. In populations at elevated risk of suicide—such as autistic people—uncovering unique or compounding factors is especially valuable, as this knowledge can guide tailored support strategies that address specific challenges and unmet needs.

To better understand correlates of suicidal thoughts in our sample, a series of binary regression models were tested. In the final model, which accounted for nearly 50% of variability for the presence of suicidal thoughts in our sample, the most robust correlates were self‐harm, depression, history of physical abuse, and a gender‐minority identity. The centrality of depression and self‐harm align with prior studies identifying mood disorders and self‐injury as significantly associated with suicidality in autistic people of all ages (Newell et al. 2023; O'Halloran et al. 2022; Storch et al. 2013) and in the general population (Mars et al. 2019; Ribeiro et al. 2016). The association with physical abuse is consistent with the wider adverse childhood experiences literature (Sahle et al. 2022) and meta‐analysis identifying childhood trauma as increasing suicidality in autistic people (O'Halloran et al. 2022). Furthermore, the association with gender‐minority identity is consistent with emerging evidence that autistic and transgender/gender‐nonconforming identities independently elevate risk for suicidality, with studies observing particularly elevated risk when they intersect (Chang et al. 2022; Lehmann et al. 2025; Mournet et al. 2024; Strauss et al. 2021).

In our model exploring the relationship between suicidal thoughts and autism‐related factors, self‐reported sensory differences and camouflaging were associated with increased likelihood of suicidal thoughts, whereas personal use of neurodiversity‐affirming language was associated with lower likelihood of suicidal thoughts. The effects of greater sensory differences and camouflaging were absent in the final model that added mental health‐related symptoms—possibly because effects operate through more proximate mental health‐related pathways or are confounded by mental health. While existing models of suicide do not include autism‐related factors, a recent study has proposed that camouflaging autistic traits may contribute to suicidality by increasing feelings of defeat (failed social struggles/feelings of powerlessness/sense of lost social status/not meeting personal goals) or entrapment (feeling trapped in aversive situations) (Cassidy et al. 2023). The finding that personal use of the term “neurodiversity” retained a significant association in the final model may reflect differences in identity or engagement with neurodiversity‐related communities that impact mental health. While encouraging, this result should be interpreted with caution. Given the cross‐sectional design of the study, we cannot determine directionality or underlying mechanisms. Further research is required to clarify if and through what pathways identification with neurodiversity is associated with resilience and may positively influence mental health outcomes. Disentangling autism‐related factors associated with suicidality remains essential for developing empirically grounded models of suicide risk in autistic populations and informing the design of tailored mental health support, including approaches informed by neurodiversity‐affirming principles (Kroll et al. 2024).

While this study has made several contributions to knowledge of suicidality among autistic youth, the findings are subject to several limitations. First, the sample comprises users of a mental health app who self‐selected to participate in the survey. Although the survey was not advertised as being about mental health (it was a general survey for autistic users, as detailed in the Methods), our sample is still likely to be enriched for mental health challenges. Participants resided in the United Kingdom and were mostly cisgender girls/women and gender minority individuals, which further limits the generalisability of our findings to all autistic youth. Our analyses included all users who believe they are autistic, which encompasses individuals with a formal diagnosis, individuals seeking a diagnosis, those who self‐identify as autistic, and those unsure whether they have been formally diagnosed. This inclusive approach is both a strength and a limitation of this work. On one hand, it aligns with community‐led, neurodiversity‐affirming research practices that recognize the barriers many people face in accessing formal diagnosis—especially girls, women, and gender‐diverse individuals (Abu‐Ramadan et al. 2025; Lockwood Estrin et al. 2021)—and therefore provides a more representative view of those who identify with the autistic experience. On the other hand, the lack of diagnostic certainty introduces heterogeneity that may obscure differences between formally diagnosed and self‐identifying subgroups and limit comparisons with studies focusing on people with a formal autism diagnosis. Second, participant age was recorded as a range rather than as an exact value. Tellmi is intended to support people aged 11–25 and users are limited to interacting with individuals within 2 years of their own age; nevertheless, the 19+ subgroup may have included a small number of participants older than 25. Future studies with larger samples could explore if correlates of suicidality differ between autistic children, adolescents, and young adults. Third, the presence of suicidal thoughts was recorded as a binary outcome, precluding analyses of their frequency, intensity, onset, or recency. Participants were not asked about suicide‐related behaviors, such as plans or attempts. Other important variables—including mental health‐related symptoms, autism‐related traits, and adverse life experiences—were similarly recorded using simplified measures. While this approach reduces participant burden while enabling exploration of various factors potentially correlating with suicidality, deeper insights could be gained by using established measures of mental health, camouflaging, and adverse experiences. Future studies could be strengthened through the use of brief self‐report measures of suicidality adapted for autistic people, such as the Suicidal Thoughts and Urges Questionnaire (STUQ) (Williams et al. 2024). Finally, data are based on anonymous self‐report. Although this approach may better capture sensitive experiences such as suicidal thoughts and traumatic experiences than informant‐based methods, responses are subject to reporting biases and item misinterpretation.

In summary, this study contributes further evidence of high rates of suicidal thoughts among young autistic people and correlates of this risk (self‐harm, depression, history of abuse, gender minority identity), while identifying a neurodiversity‐affirming identity as a potential protective factor. In line with autism community‐derived recommendations for suicide prevention and support (Moseley et al. 2026), these findings translate into clear priorities: expanding timely access to autism‐adapted mental health care for those experiencing depression and self‐harm and investing in identity‐affirming, connection‐building support to foster belonging. With high social connectedness and perceived peer support recognized as protective factors against suicidal ideation in non‐autistic youth (Gallagher et al. 2014), longitudinal studies are needed to test whether autistic youth's engagement with community‐building digital mental health services translates into sustained improvements in mental health outcomes including suicidality.

## Funding

T.L.P., E.M.W., M.P., and S.B.‐C. received funding from Autism Action (previously the Autism Centre of Excellence at Cambridge). S.B.C. also received funding from the Wellcome Trust 214322\Z\18\Z, SFARI, the Templeton World Charitable Fund, the MRC, and the Innovative Medicines Initiative 2 Joint Undertaking under grant agreement no 777394 for the project AIMS‐2‐TRIALS. This Joint Undertaking receives support from the European Union's Horizon 2020 research and innovation programme and EFPIA, Autism Speaks, Autistica, and SFARI. For the purpose of Open Access, the author has applied a CC BY public copyright license to any Author Accepted Manuscript version arising from this submission. We are grateful to Cambridge University Development and Alumni Relations (CUDAR) for anonymous donations. The funders had no role in the design of the study; in the collection, analyses, or interpretation of data; in the writing of the manuscript; or in the decision to publish the results. All research at the Department of Psychiatry in the University of Cambridge is supported by the NIHR Cambridge Biomedical Research Centre (NIHR203312) and the NIHR Applied Research Collaboration East of England. Any views expressed are those of the author(s) and not necessarily those of the funders, NIHR, or Department of Health and Social Care.

## Conflicts of Interest

K.C. and S.G. contributed as the founders and co‐CEOs of Tellmi. The other authors declare no conflicts of interest.

## Supporting information


**Table S1:** Final model tested with an expanded sample including individuals who responded to the survey item “Do you believe you are autistic” as “yes” (*n* = 365) or “unsure” (*n* = 211).

## Data Availability

The data that support the findings of this study are available on request from the corresponding author. The data are not publicly available due to privacy or ethical restrictions.

## Endnotes

^1^
The Tellmi 2022 user impact survey found that 17% of participants who responded were autistic: https://www.tellmi.help/tellmi‐autism‐project.

^2^
Only 5 of 221 (2%) individuals who answered that they were “unsure” if they believe they are autistic reported an autism diagnosis, compared to 121 of 365 (33%) individuals who answered “yes” to this question. As further justification for excluding these participants, we compared the frequency of autism‐related differences between participants who responded “yes” or “unsure”. Participants who responded “unsure” experienced all categories of autism‐related differences at lower frequencies (*χ* ^2^ tests, all *p* < 0.002).
