# Supplementary material for: Occurrence and Correlates of Suicidal Thoughts Among Young Autistic Users of a Mental Health App
Source: Autism Res. 2026 Apr 15;19(6):e70251. doi: 10.1002/aur.70251 (PMC13276680; doi:10.1002/aur.70251)
Supplement: Supplementary file 1 — Table S1: Final model tested with an expanded sample including individuals who responded to the survey item “Do you believe you are autistic” as “yes” (n = 365) or “unsure” (n = 211). [file AUR-19-0-s001.docx]

**Occurrence and Correlates of Suicidal Thoughts among
Young Autistic Users of a Mental Health App**

**Supplementary Materials**

**Supplementary Table 1.** Final model tested with an expanded sample including individuals who responded to the survey item “Do you believe you are autistic” as “yes” (n = 365) or “unsure” (n = 211).

| *Variable* | β | OR | *p* |
| --- | --- | --- | --- |
| Age range | 0.143 | 1.15 | 0.258 |
| Formal diagnosis vs Undiagnosed/Unsure | 0.393 | 1.48 | 0.168 |
| Gender minority vs. Cisgender | 0.967 | 2.63 | < 0.001 |
| Ethnic minority vs. White | -0.191 | 0.83 | 0.544 |
| Depression | 1.25 | 3.49 | < 0.001 |
| Self-harm | 2.01 | 7.45 | < 0.001 |
| Sensory differences | 0.243 | 1.28 | 0.25 |
| Camouflaging/masking | 0.351 | 1.42 | 0.075 |
| Use of term "neurodiversity" vs. Not | -0.765 | 0.47 | 0.002 |
| Physical abuse | 1.299 | 3.67 | < 0.001 |
